# Co-transfection of decorin and interleukin-10 modulates pro-fibrotic extracellular matrix gene expression in human tenocyte culture

**DOI:** 10.1038/srep20922

**Published:** 2016-02-10

**Authors:** Sunny A. Abbah, Dilip Thomas, Shane Browne, Timothy O’Brien, Abhay Pandit, Dimitrios I. Zeugolis

**Affiliations:** 1 Regenerative, Modular & Developmental Engineering Laboratory (REMODEL), Biosciences Research Building, National University of Ireland Galway (NUI Galway), Galway, Ireland; 2 Centre for Research in Medical Devices (CÚRAM), Biosciences Research Building, National University of Ireland Galway (NUI Galway), Galway, Ireland; 3 Regenerative Medicine Institute (REMEDI), Biosciences Research Building, National University of Ireland Galway (NUI Galway), Galway, Ireland

## Abstract

Extracellular matrix synthesis and remodelling are driven by increased activity of transforming growth factor beta 1 (TGF-*β*1). In tendon tissue repair, increased activity of TGF-*β*1 leads to progressive fibrosis. Decorin (DCN) and interleukin 10 (IL-10) antagonise pathological collagen synthesis by exerting a neutralising effect via downregulation of TGF-*β*1. Herein, we report that the delivery of DCN and IL-10 transgenes from a collagen hydrogel system supresses the constitutive expression of TGF-*β*1 and a range of pro-fibrotic extracellular matrix genes.

Tendons and ligaments represent the most common musculoskeletal injuries. Of the estimated 100 million musculoskeletal injuries occurring annually worldwide, 30–50% are tendon and ligament related, with an associated healthcare expenditure in excess of US$ 140 billions^1^,^2^,^3^. Unfortunately contemporary therapeutic strategies, including the gold standard autologous graft-based surgical repair, often lead to fibrotic healing with formation of adhesions, contractures and scars^4^,^5^. As a result, the healed tissue is structurally and mechanically weaker than the original tendon tissue with an increased propensity for re-injury.

The pathologic hallmark of tendon fibrosis is underlined by changes in the composition of interstitial extracellular matrix (ECM)^6^,^7^,^8^,^9^,^10^. This is associated with the failure of timely termination of key molecular processes in the normal healing cascades, presumably due to continuing presence of physical and biochemical triggers and regulators^11^. Transforming growth factor beta 1 (TGF-*β*1) is a pleiotropic growth factor that plays a central role in orchestrating fibroblast mitogenesis, proliferation and an array of molecular responses to injuries^12^. However, excessive or prolonged production of TGF-*β*1 has been directly linked to the accumulation and disorderliness of fibrillogenesis in wound healing and several fibrotic tissue diseases, including tendons^13^. Indeed, *in vitro* studies have demonstrated that tenocytes and mesenchymal in origin cells grown in the presence of TGF-*β*1 acquired a more fibroblastic phenotype with increased expression of collagen type I, collagen type III, fibronectin, elastin, laminin and proteoglycans^13^,^14^. Consistently, *in vivo* studies in tendons and other tissues have revealed that administration of TGF-*β*1 promptly triggers a range of pro-fibrotic tissue reactions^12^,^15^, whilst neutralisation effectively abrogates the development of the fibrotic response and in some cases also induces dissolution of already formed fibrotic tissues^15^. Therefore, the suppression or control of TGF-*β*1 and its pro-fibrotic activities has been identified as candidate prophylactic and therapeutic strategies in addressing fibrotic wound healing.

Proteoglycans play an important role in stabilising collagen fibres and docking of growth factors required for normal tendon function^10^, whilst pro-inflammatory and anti-inflammatory cytokines have regenerative effect in tendon healing^16^,^17^. Specifically to fibrosis, decorin (DCN), a predominant proteoglycan constituent of tendon ECM, has been shown to bind and inhibit the production of TGF-*β*1, thus modulating a number of TGF-*β*1 related ECM metabolic activities, including fibrillogenesis and collagen fibril organisation^18^,^19^,^20^. In a similar manner, the anti-inflammatory cytokine interleukin 10 (IL-10) has been shown to protect against TGF-*β*1 induced scar formation and to promote remodelling and wound healing^21^,^22^,^23^,^24^,^25^.

Given that the development of fibrosis in tendon is orchestrated through a battery of signalling molecules secreted by multiple cell types, multi-modal therapeutic interventions could potentially overcome the drawbacks of single molecule therapeutics^26^,^27^,^28^,^29^,^30^,^31^,^32^,^33^. Herein, it is hypothesised that a multi-modal and sustained delivery of DCN and IL-10 encoding plasmid DNA via a collagen type I reservoir will downregulate TGF-*β*1 and associated ECM components in primary human tendon fibroblast culture.

## Results

### Assessment of pDCN, pIL-10 and pDCN+IL-10 immobilisation and release

**Figure 1:**
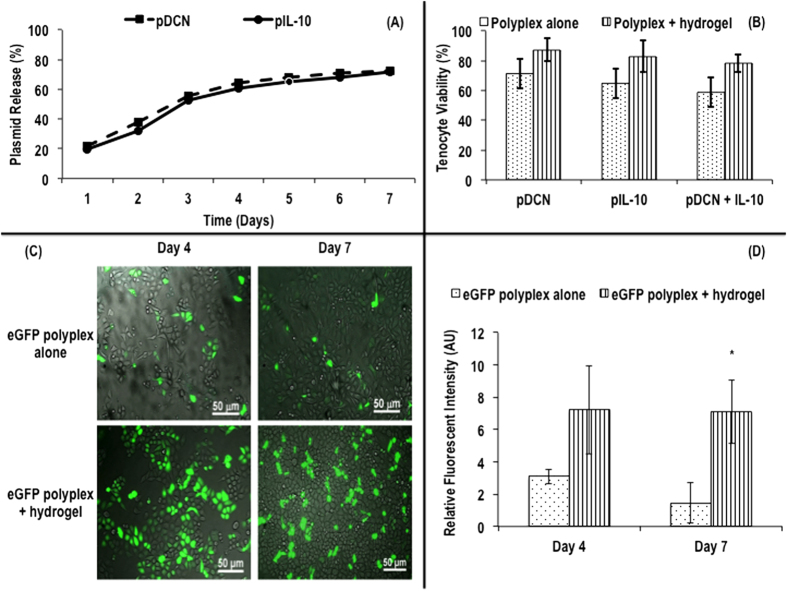
Complexed pDCN and pIL-10 release from hydrogel in PBS over seven days (**A**). No significant difference (*p* > 0.05) in % human tenocyte viability was observed after transfection with complexed plasmids (pDCN, pIL-10, pDCN+IL-10) or plasmids (pDCN, pIL-10, pDCN+IL-10) in collagen hydrogel (**B**). The use of atelocollagen gel embedment of pDNA-polymer complexes to deliver eGFP showed improved green florescent protein expression at both 4 and 7 days post transfection, when compared with conventional direct pDNA-polymer complexes exposure to cells (**C**). Relative fluorescent intensity of eGFP after transfection revealed that transgene overexpression was extended for a period of 7 days (D). Note: *indicates *p* < 0.001.

pDCN, pIL-10 and pDCN+IL-10 were embedded within the atelocollagen hydrogel to determine the plasmid release profile over a 7 day period. Within the first 24 hours, a 20% elution of immobilised pDNA into the PBS incubation buffer was observed. The total DNA elute increased to over 70% on day 4 and continued to rise gradually reaching 74% on day 7 irrespective of the specific pDNA immobilised (Fig. 1A).

The viability of the tenocytes was assessed in culture after the addition of pDNA polyplexes (e.g. pDCN, pIL-10 and pDCN+IL-10) with or without the collagen hydrogel. No statistical significance between the groups was observed (Fig. 1B).

To further characterise the influence of collagen hydrogel as a reservoir for prolonged pDNA release, an enhanced green fluorescent protein (eGFP) reporter gene was complexed and loaded in collagen hydrogel and delivered to human tenocytes. Fluorescence microscopy (Fig. 1C) and complementary relative fluorescent intensity analysis (Fig. 1D) confirmed that transgene overexpression was extended for a period of 7 days.

**Figure 2:**
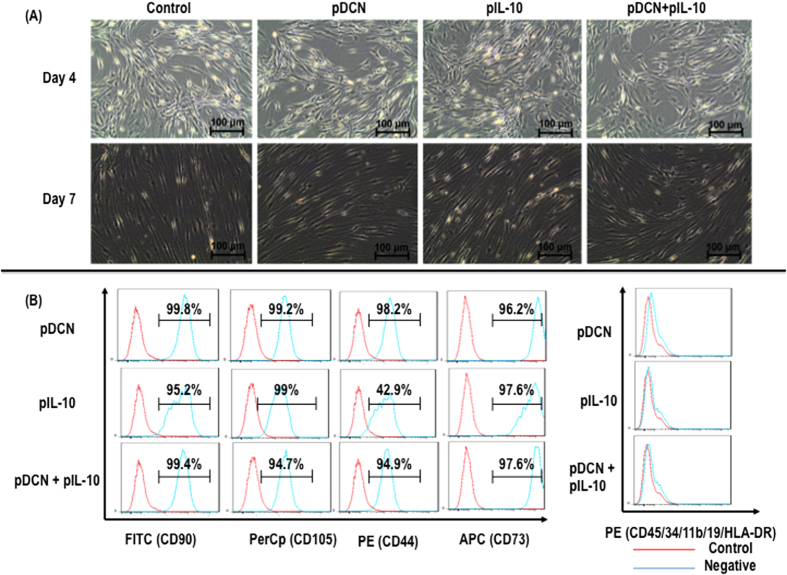
pDCN, pIL-10 and pDCN+IL-10 did not change tenocyte morphology (**A**). Flow cytometry analysis indicated that pDCN, pIL-10 and pDCN+IL-10 did not affect CD90, CD105, CD44 and CD73 expression (**B**).

Transfer of pDCN, pIL-10 and pDCN+IL-10 did not affect tenocyte morphology (Fig. 2A) and did not affect surface marker expression (Fig. 2B).

### Assessment of pDCN, pIL-10 and pDCN+IL-10 on DCN and IL-10 expression in human tenocyte culture

**Figure 3:**
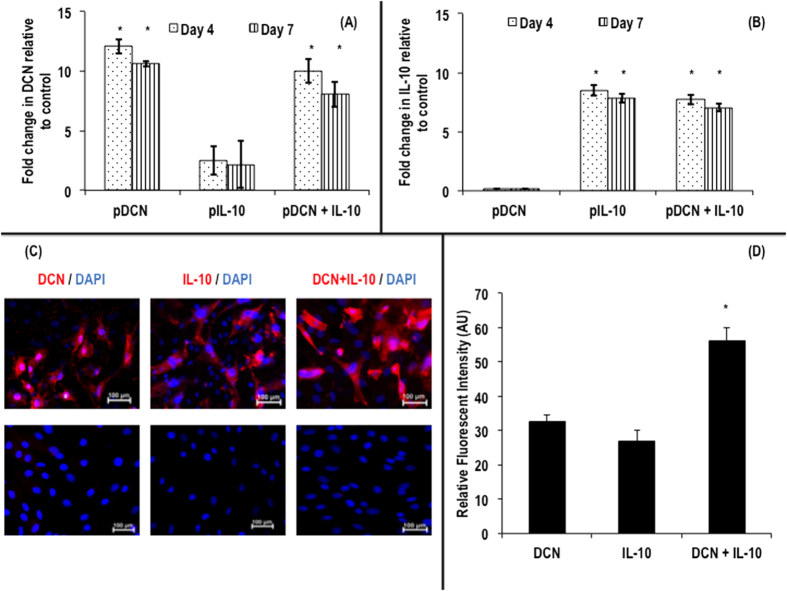
Quantitative real-time PCR analysis of DCN (**A**) and IL-10 (**B**) gene expression in cultured human tenocytes after transfection with pDCN, pIL-10 and pDCN+IL-10. pDCN and pDCN+IL-10 at both time points (day 4 and 7) resulted in significant increase (*p* < 0.001) in DCN gene expression (**A**). pIL-10 and pDCN+IL-10 at both time points (day 4 and 7) resulted in significant increase (*p* < 0.001) in IL-10 gene expression (**B**). (**C**) Supplementary immunocytochemistry analysis further confirmed increased DCN and IL-10 protein expression in transfected cells (up row) at day 7 (**C**). Relative fluorescent intensity confirmed increased DCN and IL-10 protein expression in transfected cells at day 7, with DCN+IL-10 exhibiting the highest (*p* < 0.001) increase (**D**). Note: *indicates *p* < 0.001.

Compared to baseline expression (expression levels in non-transfected tenocytes), at day 4 and at day 7, a 12.1- and a 10.7- fold increase in gene expression of DCN was observed respectively, when human tenocytes were treated with pDCN (Fig. 3A). At day 4 and at day 7, a 2.5- and a 2.2- fold increase in gene expression of DCN was observed respectively, when human tenocytes were treated with pIL-10 (Fig. 3A). At day 4 and at day 7, a 10.0- and an 8.1- fold increase in gene expression of DCN was observed respectively, when human tenocytes were treated with pDCN+IL-10 (Fig. 3A).

Compared to baseline expression (expression levels in non-transfected tenocytes), at day 4 and at day 7, a 0.1- and a 0.2- fold increase in gene expression of IL-10 was observed respectively, when human tenocytes were treated with pDCN (Fig. 3B). At day 4 and at day 7, an 8.5- and a 7.8- fold increase in gene expression of IL-10 was observed respectively, when human tenocytes were treated with pIL-10 (Fig. 3B). At day 4 and at day 7, a 7.7- and a 7.1- fold increase in gene expression of IL-10 was observed respectively, when human tenocytes were treated with pDCN+IL-10 (Fig. 3B).

Immunocytochemical staining (Fig. 3C) and complementary relative fluorescent intensity analysis (Fig. 3D) further confirmed increased DCN and IL-10 protein expression in transfected cells at day 7.

### Assessment of pDCN, pIL-10 and pDCN+IL-10 on TGF-*β*1 expression of human tenocytes

**Figure 4:**
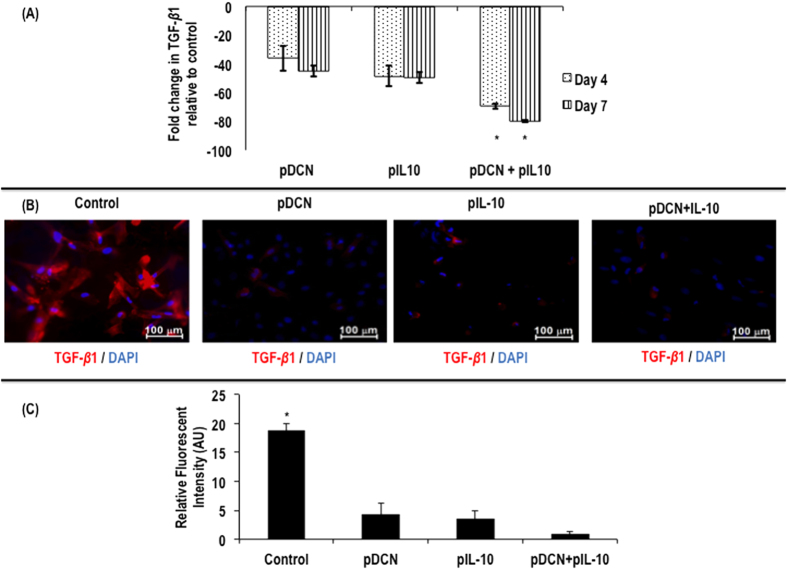
Quantitative real-time PCR analysis of TGF-*β*1 gene expression in human tenocytes following transfection with pDCN, pIL-10 and pDCN+IL-10 polyplexes delivered using a collagen hydrogel system indicate that pDCN+IL-10 is more effective (*p* < 0.001) in suppressing TGF-*β*1 gene expression (**A**). Supplementary immunocytochemistry analysis for TGF-*β*1 further confirmed the increased efficacy of pDCN+IL-10 over pDCN and pIL-10 in supressing TGF-*β*1 expression (**B**). Relative fluorescent intensity analysis confirmed the effectiveness of pDCN, pIL-10 and pDCN+pIL-10 in significantly (*p* < 0.001) supressing TGF-*β*1 expression (**C**). Note: *indicates *p* < 0.001.

Expression of TGF-*β*1 gene was reduced at day 4 by ~36.00% and at day 7 by ~45.10 in pDCN and by ~48.50% at day 4 and ~49.60% at day 7 in pIL-10 transfected human tenocytes (Fig. 4A). Combined treatment with pDCN+IL-10 led to pronounced reduction in TGF-*β*1 gene expression, with ~69.40% and ~79.70% relative to non-transfected cells at day 4 and 7 respectively (Fig. 4A). At day 7, immunofluorescent staining (Fig. 4B) and complementary relative fluorescent intensity analysis (Fig. 4C) confirmed the downregulation of TGF-*β*1.

### Assessment of pDCN, pIL-10 and pDCN+IL-10 on collagen type I, collagen type III, fibronectin and elastin gene expression in human tenocyte culture

**Figure 5:**
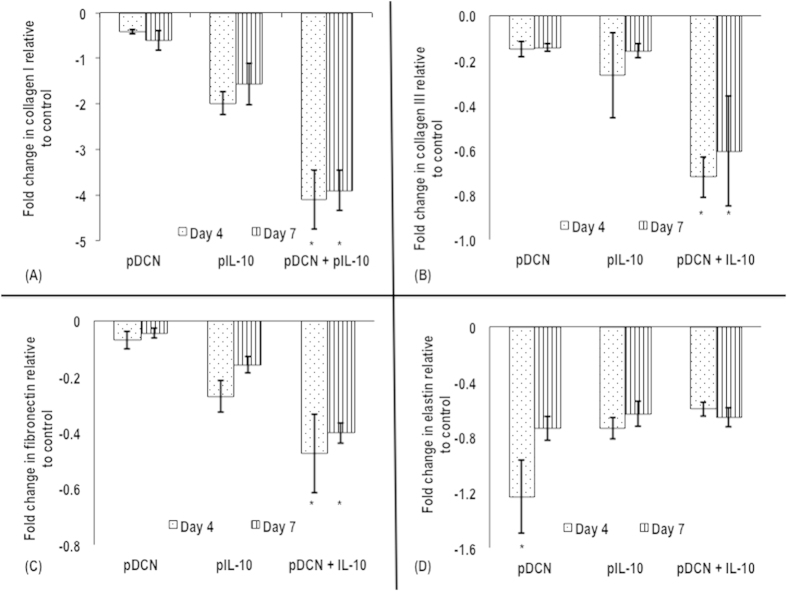
Quantitative real-time PCR analysis of collagen type I (**A**), collagen type III (**B**), fibronectin (**C**) and elastin (**D**) as a function of pDCN, pIL-10 and pDCN+IL-10 polyplexes and time in culture (4 and 7 days). pDCN+IL-10 significantly (*p* < 0.001) suppressed collagen type I (**A**), collagen type III (**B**) and fibronectin (**C**) by at least 2-fold more than single pDCN or pIL-10 treatments. Similar inhibitory effect was not seen in the gene expression of elastin (**D**). Note: *indicates *p* < 0.001.

pDCN and pIL-10 decreased expression of collagen type I (Fig. 5A), collagen type III (Fig. 5B), fibronectin (Fig. 5C) and elastin (Fig. 5D) genes in transfected tenocytes, when compared to untreated cells (control group) at both 4 and 7 days. pDCN+IL-10 not only suppressed collagen type I (Fig. 5A), collagen type III (Fig. 5B) and fibronectin (Fig. 5C), when compared to untreated cells (control group), but also it was by at least 2-fold more effective than pDCN or pIL-10 alone treatments at both time points (days 4 and 7). However, such inhibitory effect was not seen in the gene expression of elastin (Fig. 5D).

## Discussion

Disproportionate synthesis and deposition of ECM in response to trauma and persistent inflammation lead to tissue fibrosis and consequent loss of tissue integrity, pain, inelastic scars, intercepting fibrillar regeneration and interrupted functional tenogenesis^34^. TGF-*β*1 is known to be one of the key mediators in driving fibrosis through canonical (e.g. SMAD 2 and 3) and non-canonical (e.g. MAPK) pathways^35^. *In vitro* studies have demonstrated that tenocytes and mesenchymal progenitors grown in the presence of TGF-*β*1 acquired a more fibroblastic phenotype, with increased expression of collagen type I, collagen type III, fibronectin, elastin, laminin and proteoglycans^13^,^14^. Consistently, *in vivo* studies in tendons and other tissues, have revealed that administration of TGF-*β*1 proteins promptly triggers a range of pro-fibrotic tissue reactions^12^,^15^. Several vector-based gene therapy studies have targeted TGF-*β*1 or its mediators, in order to knockdown scar formation and promote tenogenesis^36^,^37^,^38^. However, clinical translation of vector-based gene therapy products pose major safety and toxicology concerns^17^.

In the present study, we have developed a collagen-based hydrogel for delivery pDCN, pIL-10 or pDCN+IL-10 as a means to modulate expression of TGF-*β*1 and associated ECM mediators of tissue fibrosis. The rationale of using a collagen-based scaffold is based on the fact that collagen serves as bioactive depot and acts as a natural entrapment for binding of growth factors and proteoglycans, promoting tissue ingrowth^39^. We recognise that the high binding affinity of biglycan and fibromodulin to TGF-*β* has been previously harnessed to sequester TGF-*β* signalling activities *in vitro*^40^,^41^. Indeed, whereas DCN and biglycan only sequesters the active form of TGF-*β*, fibromodulin is able to bind to even the latent form of TGF-*β*, resulting in a more comprehensive inhibition. In theory, fibromodulin gene therapy could represent a more robust control of TGF-*β* mediated fibroplasia *in vivo*. However, given the multiplicity roles of this growth factor in wound healing, excessive inhibition of TGF-*β* could be deleterious to the innate process of wound healing. Thus, herein DCN and IL-10 were chosen, as their anti-TGF-*β*1 efficacy has been well established in the literature (e.g. DCN^42^,^43^,^44^,^45^, IL-10^21^,^46^,^47^,^48^).

Sustained release of the plasmids was observed over 7 days period, without any negative effects on tenocyte viability, morphology and surface marker expression. Although polyplexes can induce cytotoxicity due to molecular weight, charge and degradation products^49^, similar to our results, loading polyplexes within a collagen hydrogel prevents polyplex-induced cytotoxicity^50^,^51^,^52^.

Delivery of pDCN and pIL-10 did not significantly elevate transcription of IL-10 and DCN respectively, whilst delivery of pDCN+IL-10 induced comparable levels of DCN and IL-10 expression, illustrating no competitive or inhibitory mode of action. When the efficacy of pDCN and pIL-10 against TGF-*β*1 was assessed, both were found to be proportionately effective, however pDCN+IL-10 was more effective than the individual plasmids. The co-expression of pDCN+IL-10 against TGF-*β*1 was also verified with downregulation in collagen type I, collagen type III and fibronectin gene expression. Previous studies have demonstrated that ECM genes are regulated by TGF-*β* activity^53^ and the magnitude of difference depends on the cell type^54^,^55^,^56^. With respect to elastin expression, the most profound difference was observed in pDCN. DCN has been shown to be involved in early stages of elastino-genesis^57^,^58^. Particularly, action of DCN via TGF-*β* sequestration destabilises ECM by accelerating tissue destruction^59^,^60^. On the other hand, IL-10 in presence of TGF-*β* is known to up-regulate elastin gene expression^61^. Hence, we believe that simultaneous delivery of pDCN+IL-10 modulates the opposing functions of DCN, leading to lower elastin gene expression at transcriptional level. These observations, not only illustrate the independent mode of action of the plasmids, but also corroborate previous publications, where co-delivery of therapeutics/biologics was demonstrated to be superior to mono-domain approaches^62^,^63^,^64^,^65^,^66^,^67^.

Although virus-based direct transfer of gene sequences with remarkable efficiency has been demonstrated, safety concerns including the risk of cytotoxicity, immunogenicity and teratogenicity have perennially limited the clinical efficacy of viruses as vectors in gene therapy^68^. Despite the rather expansive preclinical data generated on adenovirus based gene therapies, inflammatory reaction to adenovirus vectors accounted for the first human fatality in gene therapy^69^,^70^. Given that integration of viruses into human genome preferentially occurs at transcriptionally active regions, concerns of mutagenesis leading to malignancies remains clinically relevant with viral vector strategies^68^. Therefore, non-viral vector platforms^71^,^72^,^73^,^74^, such as the one described in the present study, promise to address these safety concerns.

## Conclusions

In the present study, pDCN+IL-10 delivered through a collagen hydrogel system enhanced suppressive effect on TGF-*β*1 expression and related ECM genes (collagen type I, collagen type III and fibronectin) in human tenocyte culture. These findings have important therapeutic implications in fibrosis and further corroborate the notion for cocktail treatments.

## Materials and Methods

### Cell culture

Primary human tenocytes were purchased from Cambridge Biosciences (Cambridge, UK). Primers were purchased from Eurofins MWG GmbH (Ebersberg, Germany). TransIT^®^-LT1 transfection reagent was purchased from Mirus Bio (Madison, WI, USA). Polyclonal antibodies (raised in rabbit) against human TGF-*β*, DCN and IL-10 were purchased from Abcam (Abcam Inc., Cambridge, MA, USA). All media and media supplements were obtained from Gibco/Invitrogen (Bio-sciences, Dublin, Ireland). Laboratory consumables were obtained from Sigma-Aldrich (Arklow, Ireland), unless otherwise stated.

### Plasmid polyplex formation and gene transfection

TransIT^®^-LT1 transfection reagent was complexed with plasmid DNA (eGFP, DCN or IL-10 as appropriate) following manufacturer’s instructions. Human IL-10 cDNA clone inserted in plasmid DNA (pCMV6-XL5, Cat. No. SC300099) and human DCN cDNA clone inserted in plasmid DNA (pCMV6-AC, Cat. No. SC320831) were purchased from OriGene Technologies (Rockville, MD, USA). Briefly, TransIT^®^-LT1 transfection reagent was mixed with pDNA at a variety of TransIT^®^-LT1 transfection reagent amino–DNA phosphate charge (N:P) ratios (1:1, 2:1, 5:1, 10:1 etc.) in serum-free media and were allowed to form complexes for 20 minutes. Human tenocytes were cultured in DMEM supplemented with 10% foetal bovine serum (FBS; Invitrogen), 1% pen-strep (Invitrogen) and 0.2% L-Glutamine (Invitrogen, Bio-sciences, Dublin, Ireland) at 37 °C in a humidified atmosphere of 5% CO_2_. Tenocytes were plated on 6-well plates in the presence of atelocollagen gels containing pDNAs complexes encoding IL-10 and/or DCN. Cellular metabolic activity was analysed on days 4 and 7 using the alamarBlue^®^ assay, as per manufacturer’s protocol (Life Technologies™, Bio Sciences, Dublin, Ireland).

### Atelocollagen gel embedment of pDNA-polymer complexes

Freeze-dried type I atelocollagen, extracted from bovine Achilles tendon as has been described previously^75^, was sterilised in ethanol and reconstituted in diluted acetic acid (1:1000 acetic acid in double distilled water) at a concentration of 0.15%. Eight volumes of the 0.15% collagen solution were mixed with 2 volumes of pDNA-polymer complexes (previously determined ratio) in serum-free culture media and 0.34 M NaOH. The solution was kept in ice to avoid premature self-assembly. Self-assembly occurred at 37 °C.

### Plasmid DNA release

The release of the pDNA polyplexes from the atelocollagen hydrogel was characterised using the PicoGreen™ assay Kit (Invitrogen Life Technologies, Carlsbad, CA, USA) according to the manufacturer’s protocols. Briefly, the hydrogels containing pDNA polyplexes were prepared as described above in a 6-well plate. The loaded gels were left standing for 3 hours at room temperature to allow for complete gelation. Subsequently, gels were transferred to 6-well plates. Differences in the release profiles of two different pDNAs (IL-10 and DCN) from the gel were investigated. To this, phosphate buffered saline (PBS) was added to the wells and incubated at 37 °C (pH 7.0). At each time point, the wash media was removed and replaced with an equal volume. At the end of the experiment, the DNA content of the wash media was quantified using the PicoGreen™ assay and the cumulative release of DNA from the gels was calculated following comparison with a standard curve.

### RNA extraction

Extraction of RNA from the human tenocytes exposed to gels with or without pDNA was performed according to the manufacturer’s protocols. Briefly, cells were detached from 6-well plates using trypsin treatment. 1 ml of TRI Reagent^®^ (Applera Ireland, Dublin, Ireland) was added to each sample and incubated for 5 minutes at room temperature. Chloroform phase separation was performed and total RNA was purified using an RNeasy^®^ kit (Qiagen), following protocols recommended by the manufacturer. The total quantity and purity of RNA was evaluated with an ultraviolet spectrophotometer (NanoDrop ND-1000 Spectrophotometer; NanoDrop Technologies, Wilmington, USA).

### Reverse transcription polymerase chain reaction (RT-PCR)

Reverse transcription (RT) reactions were carried out and monitored with the TaqMan^®^ Real-time Gene Expression Mastermix (Applied Biosystems, Foster City, CA, USA) and an ABI 7000 sequence detection system (Applied Biosystems^®^). Details of specific primers are provided in Table 1. The primers were designed and their specificity checked via primer-BLAST (www.ncbi.nlm.nih.gov). Gene transcription was inferred from calibration samples and normalised in relation to transcription of the housekeeping gene glyceraldehydes-3-phosphate dehydrogenase (GAPDH). The 2^ΔΔ−Ct^ method was used to calculate relative expression for each gene.

### Immunofluorescence assay

Human tendon cells were seeded onto sterilised microscope slide coverslips at a density of 10^4^/mL and cultured in a 6-well plate as described above. At predetermined time points, the cells on coverslips were washed in PBS and fixed in 2% paraformaldehyde-PBS for 20 minutes, permeabilised in 0.5% Triton X-100 in PBS and blocked in 1% bovine serum albumin for 30 minutes at room temperature. Cells were subsequently incubated overnight with appropriate primary antibody (1:200), followed by PBS washing and detection with TRITC-conjugated secondary antibody (1:300). The nuclei were counterstained with 4’,6-diamidino-2-phenylindole (DAPI). Quantitative evaluation of relative fluorescence intensity was conducted on digital fluorescent images of cells captured using an Olympus IX81 microscope at 10× and 20× magnifications.

### Flow cytometry

Flow cytometry analysis was used for representative tendon-derived cell surface markers^2^,^76^,^77^. The cells were detached and washed twice in PBS. The cells were re-suspended in FACS buffer (PBS, 2% FBS and 0.1% NaN_3_). Approximately, 2.5 × 10^5^ cells were incubated with anti-human primary monoclonal antibodies CD90, CD105, CD44, CD73, CD45, CD34, CD11b, CD19, and HLA-DR (Miltenyi Biotec, Bergisch-Gladbach, Germany). Data were acquired by BD FACS Canto (BD Biosciences, San Jose, CA) FACS Calibur flow cytometer and analysed by FlowJo software (TreeStar Inc., OR, USA).

### Statistical analysis

Results are expressed as mean ± standard deviation and analysis of variance (ANOVA) followed by Tukey’s multiple comparison tests were used to determine statistical significance. Data were considered statistically significant at *p* < 0.05. In all studies, the minimum sample size was 4.

## Additional Information

**How to cite this article**: Abbah, S. A. *et al.* Co-transfection of decorin and interleukin-10 modulates pro-fibrotic extracellular matrix gene expression in human tenocyte culture. *Sci. Rep.* **6**, 20922; doi: 10.1038/srep20922 (2016).

## Figures and Tables

**Table 1:** Set of primers used for real-time quantitative PCR.

Gene	Forward Primer	Reverse Primer
Collagen I	5′ TGACCTCAAGATGTGCCACT 3′	5′ ACCAGACATGCCTCTTGTCC 3′
Collagen III	5′ GCTGGCATCAAAGGACATCG 3′	5′ TGTTACCTCGAGGCCCTGGT 3′
Fibronectin	5′ CGCAGCTTCGAGATCAGTGC 3′	5′ TCGACGGGATCACACTTCCA 3′
Elastin	5′ CTGGCGTGCCTGGGGCAATTCCTG 3′	5′ TTGCGGCTAGGGTCTCCGAGGTC 3′
TGF-*β*1	5′ TGAAGTGGTCTTTTGACG 3′	5′ GTTGGTTGTAGAGGGCAAGG 3′
DCN	5′ CGCCTCATCTGAGGGAGCTT 3′	5′ TACTGGACCGGGTTGCTGAA 3′
IL-10	5′ AGAACCT GAAGACCCTCAGGC 3′	5′ CCACGGCCTTGCTCTTGTT 3′
GAPDH	5′ CCATGAGAAGTATGACAACAGCC 3′	5′ CCTTCCACGATACCAAAGTTG 3′
